# Socially Disruptive Technologies, Contextual Integrity, and Conservatism About Moral Change

**DOI:** 10.1007/s13347-022-00578-4

**Published:** 2022-08-30

**Authors:** Ibo van de Poel

**Affiliations:** Section Ethics and Philosophy of Technology, Department of Values, Technology & innovation, School of Technology, Policy & Management, TU Delft, Jaffalaan 5, 2628 BX Delft, the Netherlands

**Keywords:** Technological change, Disruptive technologies, Contextual integrity, Technomoral change, Value change, Ethics

## Abstract

This commentary is a response to *Contextual Integrity as a General Conceptual Tool for Evaluating Technological Change* by Elizabeth O’Neill (*Philosophy & Technology* (2022)). It argues that while contextual integrity (CI) might be an useful addition to the toolkit of approaches for ethical technology assessment, a CI approach might not be able to uncover all morally relevant impacts of technological change. Moreover, the inherent conservatism of a CI approach might be problematic in cases in which we encounter new kinds of morally problematic situations, such as climate change, or when technology reinforces historically grown injustices.

Socially disruptive technologies might be understood as technologies that disrupt existing practices and institutions (cf. Hopster, 2021). They are not just very impactful, but they imply a long-term and difficult to reverse change in social rules and entrenched norms. They change the norms we follow in certain contexts and by which we judge the behavior of ourselves and others. A popular example is the birth control pill, which allegedly led to — or at least contributed to — a change in sexual morality (e.g., Swierstra, 2013). Such long-term normative changes might be hard but not impossible to reverse, as the recent US Supreme Court ruling on the right to abortion suggests.

Elizabeth O’Neill proposes a “step-by-step procedure for assessing whether a technological change is likely to facilitate social or moral disruption and, ultimately, whether the technological change is likely to advance or threaten our most important ends” (O’Neill, 2022: 2). She does so by broadening Helen Nissenbaum’s (2004) concept of contextual integrity (CI); the core idea is that entrenched contextual norms in society are functional in serving shared ends. Since new technologies may disrupt such entrenched norms, they may also hamper the possibility to attain important shared ends. In the proposed procedure, the evaluator first evaluates whether entrenched norms are likely to be disrupted by technological change, which would constitute a *prima facie* CI violation. However, such a prima facie CI violation need not be a *genuine* CI violation as entrenched norms sometimes may hinder rather than advance important shared ends. In the final step, the evaluator decides whether the genuine CI violation is also *problematic*.

The procedure that O’Neill proposes is an important contribution to the literature on disruptive technologies and technomoral change. It offers handles for describing and normatively judging new technologies and their effects on norms and values. Recently, there has been a lot of attention for phenomena like technomoral change, moral revolutions, and value change (Hopster et al., 2022; Melnyk, 2022; Nickel et al., 2022; Swierstra, 2013), but this literature has not yet taken up the challenge of developing frameworks for also normatively judging norm or value change (Van de Poel, 2022). For example, Boenink et al. (2010) reject what they call moral presentism (basing once’s moral analysis solely on current values and norms), but they do not provide an alternative for normatively judging technomoral change. O’Neill's approach is more conservative. She follows Nissenbaum who has suggested that “the entrenched normative framework represents a settled rationale for a certain context that we ought to protect unless powerful reasons support change” (Nissenbaum, 2004: 217). In O’Neill’s proposal, such powerful reasons would primarily refer to shared ends, although she recognizes that these might also change.

While I believe that it is a useful idea to use CI for normatively judging technological change, I also think the approach has its limitations. There seem to be some categories of technological change for which a CI approach might be problematic. I start with outlining three of such cases. I then point at two underlying reasons why I think these cases cannot be properly analyzed with the help of CI: some moral problems do not constitute a CI violation and the inherent conservatism in the approach.

Let me start with three types of cases of technological change that I believe a CI approach might not adequately handle from a normative point of view: (1) some cases of environmental degradation, (2) some cases of distributive justice, and (3) cases that transgress the boundaries of specific contexts.

Some technologies are disruptive with respect to the natural environment but without necessarily being *socially* disruptive. Technologies like coal plants may contribute to environmental damage and to climate change, and they may ultimately endanger the future of humanity. In this sense, they may be disruptive but they are not — or at least not necessarily — also *socially* disruptive, in the sense of disrupting entrenched norms. For some energy technologies, there were no social or moral norms yet when they were introduced, or they simply reinforced existing ones. This is understandable because many of the current (contextual) normative frameworks were developed in times that environmental degradation was not yet a major issue (cf. Jonas, 1984). In such cases, it is questionable whether a CI-based normative analysis is adequate. The point is not only that there might not be a CI violation in such cases, but also that the inherent conservatism in the CI approach is in this type of cases counterproductive.

Another type of cases is those where technologies have major distributive effects. As authors like Winner (1980), Noble (1984), and Feenberg (1991) have suggested, technologies may have major distributive effects not only on the distribution of (social) goods but also on the distribution of power. As O’Neill recognizes, the CI approach has been criticized for ignoring issues of power when it comes to creation of (entrenched) norms, and she distinguishes therefore between prima facie and genuine CI violations. But what about cases in which technology reinforces existing power imbalances and injustices but *without* violating entrenched norms? It would seem that such cases remain under the radar in the proposed approach. Still, such cases would seem manifold when it comes to certain digital innovations, and they are potentially disruptive not necessarily in the sense of disrupting existing norms but in the sense of creating or reinforcing morally problematic power imbalances (Van de Poel et al., 2022).

A third category of cases is where technology affects multiple contexts. An example are social media that affect contexts like friends interacting, the workplace, and public debate. Therefore, O’Neill suggest that “when evaluating social media sites, we have reason to consider their relationship to entrenched normative elements in multiple contexts” (O’Neill, 2022: 15). This is true, but it seems to miss an important point. The point is that social media tend to *blur* the distinction between different social contexts or what Walzer (2008) has called spheres of justice. It seems to me that some of the problematic phenomena we are now experiencing on social media are due to the fact that they do not clearly belong to one social context, so that people are confused what contextual norms to apply to them. The normative issue at stake here, however, cannot be solved by just looking at existing contextual norms and how they serve currently shared ends. First, social media might require new contextual norms, quite distinct from existing ones. Second, there is the deeper question whether we should want technologies to blur the distinctions between social spheres or that we believe that such distinctions are somehow crucial for human well-being and social justice (Nagenborg, 2009; Van de Poel et al., 2022).

These three examples suggest, I think, two more general limitations of a normative approach for judging new technology based on CI. First, sometimes technological change may be considered problematic even if it does not disrupt entrenched norms and therefore does not constitute a prima facie CI violation. This may be the case, as my first two examples suggest, if technological change causes environmental damage or privileges the already powerful. Such cases would remain under the radar in the proposed approach because there is no prima facie CI violation.

The other limitation has to do with the inherent conservatism in the approach. The cases show, I think, two reasons why this conservatism may be problematic. One is that current practices and contexts may be morally problematic (or sometimes even outright immoral), as recognized by O’Neill. The other is that entrenched elements of normative life sometimes might need to change because we encounter *new* types of moral problems, for which entrenched norms are dysfunctional. This seems to me the case in the first and third example. Large-scale environmental problems like climate change and the blurring of social spheres by, e.g., social media are relatively new problems (in the history of mankind), and both are, at least in part, caused by new technology. They create thus new types of morally problematic situations which may require new norms and values to adequately deal with them (van de Poel & Kudina, 2022).

At least in some cases, the conservatism inherent in a CI approach therefore seems problematic. Still, CI proponents might maintain that in general such conservatism is appropriate, or better: that we should embrace some version Chesterton’s fence as O’Neill suggests: “if we do not have a good understanding of why an entrenched element of our normative life exists, we should be cautious as we go about modifying or eliminating it, because it may have been implemented or may have arisen and been maintained because it serves a function with respect to shared ends” (O’Neill, 2022: 19).

This effectively proposes a precautionary principle when it comes to changing entrenched norms and accepting disruptive technological change. I am not unsympathetic to such a precautionary approach, but two points are worth pointing out. First, even if one subscribes to a form of functionalism about norms, norms may survive the test of time for many more reasons than being functional to shared ends; they may also be functional to the powerful, or to the existing social order, and be deeply immoral. Second, Chesterton’s fence protects us from changing entrenched norms too easily, but there are situations in which the bigger danger is in not changing entrenched norms quicky enough to address newly arisen moral problems. Climate change would seem a case in point, where some entrenched norms need no protection.

O’Neill might reply that her approach can deal with such “exceptions” because ultimately normative assessment in her approach is based on shared ends. But we might question how helpful the idea of shared ends really is as ultimate yardstick for judging technological change. O’Neill uses “ends” as “a term of art to refer to an agent’s full set of evaluative attitudes” (O’Neill, 2022: 5). The advantage of this broad understanding of ends is that the evaluator can have recourse to a wide range of normative considerations in normatively judging technological change. A disadvantage is that it seems to suggest that all possible evaluative attitudes are normatively on a par. However, we know that evaluative attitudes may be mistaken, and even if they are not mistaken, they may be overridden by other, more important, moral considerations. The question is thus whether ends really are the right kind of normative category for ultimate moral judgements about new technologies. An alternative would, for example, to judge technologies by whether they support important human capabilities, as suggested by the capability approach (Robeyns, 2017). Capability scholars have suggested that such capabilities may be a more proper normative category for judgments about human well-being than, for example, desires or preferences (e.g., Nussbaum, 2000). Moreover, since the realization of capabilities depends on conversion factors, they may be formulated in a context-sensitive way. My point is not that capabilities are necessarily the right kind of normative category to judge technological change but rather that the notion of ends that O ‘Neill currently proposes might be too broad and vague to do any real normative work.

To conclude, I think O’Neill makes an important contribution by proposing CI as a tool for evaluating technological change. I think she is right that as part of procedures for, for example, ethical technology assessment (Palm & Hansson, 2006), we should evaluate the effects of technological change on entrenched norms and on CI. However, I doubt whether a CI approach can uncover all morally relevant impacts of technological change and that its inherent conservatism is always laudable.

## Data Availability

Not applicable.
